# ﻿The Oriental fungus-feeding genus *Azaleothrips* Ananthakrishnan, 1964 from China with one new species and four new records (Thysanoptera, Phlaeothripidae, Phlaeothripinae)

**DOI:** 10.3897/zookeys.1183.113182

**Published:** 2023-11-09

**Authors:** Lihong Dang, Yaya Li, Laurence A. Mound, Gexia Qiao

**Affiliations:** 1 School of Bioscience and Engineering, Shaanxi University of Technology, Hanzhong, 723000, China Shaanxi University of Technology Hanzhong China; 2 Australian National Insect Collection, CSIRO, PO Box 1700, Canberra, ACT 2601 Australian National Insect Collection, CSIRO Canberra Australia; 3 Key Laboratory of Zoological Systematics and Evolution, Institute of Zoology, Chinese Academy of Sciences, No. 1 Beichen West Road, Chaoyang District, Beijing 100101, China Key Laboratory of Zoological Systematics and Evolution, Institute of Zoology, Chinese Academy of Sciences Beijing China; 4 College of Life Science, University of Chinese Academy of Sciences, No. 19, Yuquan Road, Shijingshan District, Beijing 100049, China University of Chinese Academy of Sciences Beijing China

**Keywords:** Identification key, mycophagous, *Phlaeothrips*-lineage, taxonomy

## Abstract

*Azaleothrips*, a genus of fungus-feeding Phlaeothripinae , is easily recognized by the complex sculpture on the body surface. It is species-rich in the Oriental region, with 10 species here recognized from China, including *A.sphaericus***sp. nov.** and four new records. An illustrated key to the species from China is provided.

## ﻿Introduction

**Figures 1–7.**
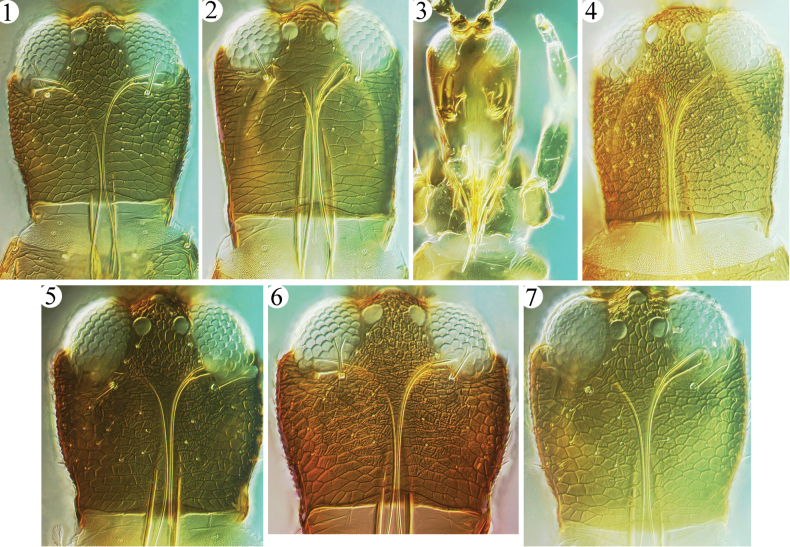
Heads of *Azaleothrips* species **1***A.formosae***2***A.laevigatus***3***A.laevigatus*, ventral view of head **4***A.sphaericus* sp. nov. **5***A.lepidus***6***A.siamensis***7***A.templeri*.

**Figures 8–14.**
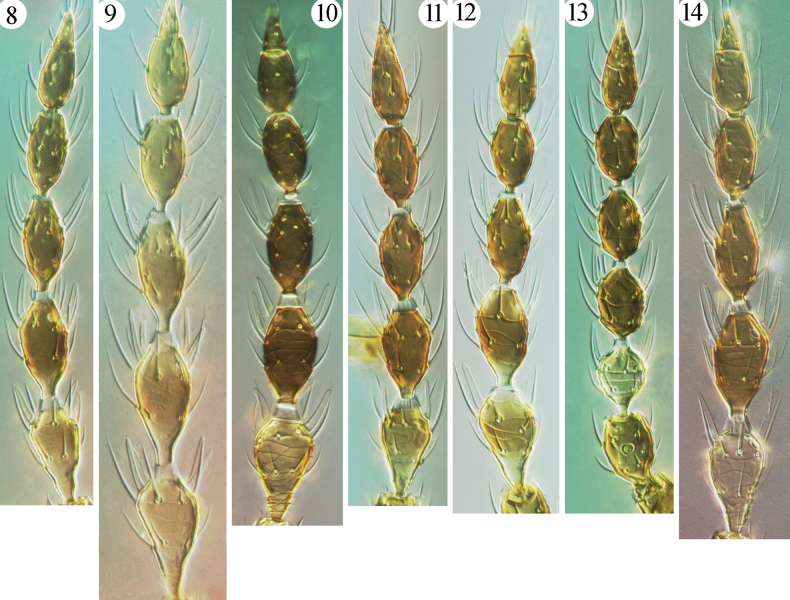
Antennae of *Azaleothrips* species **8***A.formosae***9***A.laocai***10***A.laevigatus***11***A.moundi***12***A.siamensis***13***A.sphaericus* sp. nov. **14***A.templeri*.

**Figures 15–18.**
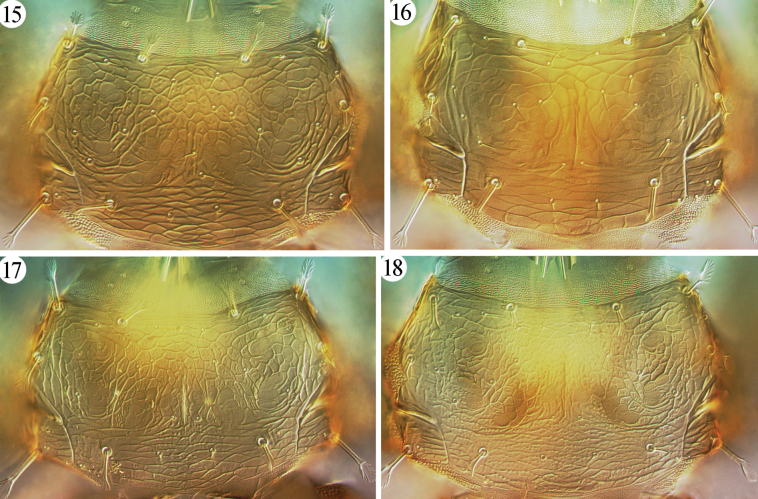
Pronotum of *Azaleothrips* species **15***A.formosae***16***A.laevigatus***17***A.lepidus***18***A.siamensis*.

**Figures 19–23.**
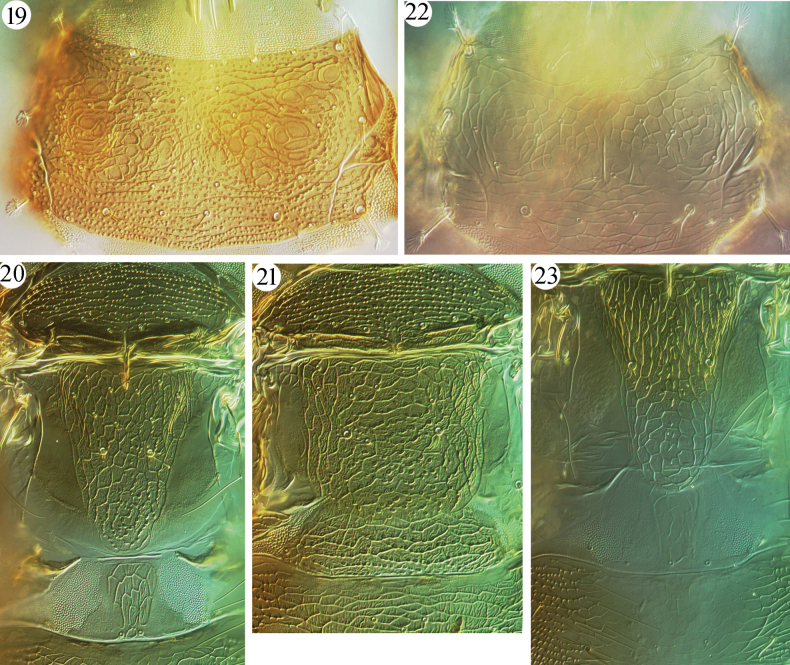
*Azaleothrips* species **19–21***A.sphaericus* sp. nov. **19** pronotum **20** mesonotum, metanotum, and pelta of macropteran female **21** mesonotum, metanotum, and pelta of micropterous female **22–23***A.templeri***22** pronotum **23** metanotum and pelta, macropterous female.

The fungus-feeding species in Phlaeothripinae belong to the *Phlaeothrips*-lineage and are usually found in dry, dead leaves, twigs, branches, and grasses (Mound and Marullo 1996). Of the 32 genera in this group recorded from China (Dang et al. 2014), *Azaleothrips* is easily recognized by the following combination of characters: body surface strongly reticulate, with many wrinkles or tubercles in lines (Figs 1–7); major setae short and broadly expanded at apex (Figs 15–19); postocular setae close together and placed near inner margin of eyes; maxillary stylets retracted to eyes and medially close together. This genus was treated as a member of the *Idiothrips* group, with three genera, *Idiothrips*, *Stegothrips*, and *Strepterothrips* (Okajima 1976), of which the first two are unknown in China but easily distinguished from *Azaleothrips*, as indicated by Okajima and Masumoto (2014). *Strepterothrips*, of which only *S.orientalis* of the 15 known species is recorded from Taiwan, is closely related to the genus *Azaleothrips* (Okajima 1995; ThripsWiki 2023), but it can be distinguished by having one sense cone on antennal segment III and a well-developed fore tarsal ventral hamus (Okajima and Masumoto 2014). In addition, *Strepterothrips* species have seven antennal segments, with the morphological VIII joined to VII without a suture, are usually wingless, and have antennal segment II obviously larger than segment I. In comparison, *Azaleothrips* has antennal segment VIII distinct from VII and with at least a complete suture (Figs 8–14), is usually macropterous, and antennal segment II is regular. A related genus, *Stictothrips*, also shares the complex body sculpture and fan-shaped major setae with *Azaleothrips*, but it has the fore wings curiously constricted and twisted (Mound and Tree 2015).

In the process of identifying slides of *Azaleothrips* from China, two species were recognized, *A.laevigatus* and *A.dentatus*, which seem to be unusual in having the dorsal view of body not heavily reticulate (Fig. 2), the maxillary stylets and mouth cone elongate (Fig. 3), and the major setae relatively longer. These two are similar to females of the genus *Ablemothrips*, with which they share the following characters: postocular setae close, antennal segments VII–VIII fused, antennal segments III–IV with three and four sense cones respectively, and forewings slender and weakly medially constricted. The only character state that distinguishes *Ablemothrips* from the above two species is the sexual dimorphism in the position of the postocular setae, which are widely separated in males of all three *Ablemothrips* species(Okajima 1999). This condition is not recorded in any *Azaleothrips*.

Among the 35 species of *Azaleothrips*, two species groups have been recognized: the *amabilis* species group with nine species, and the *moundi* species group with 26 species (Okajima and Masumoto 2014). In China, only two species have been recorded (Han 1992; Dang et al. 2014), *A.moundi* and *A.siamensis*, but three more were described from Taiwan by Okajima and Masumoto (2014): *A.formosae*, *A.taiwanus*, and *A.atayal*.

The objective here is to recognize species of the Oriental genus *Azaleothrips* in the first author’s thrips collection, to describe a new species, and to provide an illustrated key to 10 Chinese *Azaleothrips* species, including four new records for *A.laevigatus*, *A.laocai*, *A.lepidus* and *A.templeri*. The new species, *A.sphaericus* sp. nov., obviously belongs to the *moundi* species group because it has two sense cones on antennal segment III (Fig. 13), closely fused VII–VIII (Fig. 13), no fore-tarsal tooth in either sex, an expanded S2 on male tergite IX (Fig. 28), and many short discal setae on the pronotum and metanotum (Figs 19–21).

## ﻿Materials and methods

The descriptions and photomicrographs were produced from slide-mounted specimens under a Nikon Eclipse 80i microscope with a Leica DM2500 camera and using differential interference contrast illumination. These images were processed with Automontage and Photoshop v. 7.0. The abbreviations used for the pronotal setae are as follows: am – anteromarginal, aa – anteroangular, ml – midlateral, epim – epimeral, pa – posteroangular. The unit of measurement in this study is the micrometre. Most specimens studied here are available in the School of Bioscience and Engineering, Shaanxi University of Technology (**SNUT**), Hanzhong, China, the Australian National Insect Collection (**ANIC**), Canberra, Australia, and the National Zoological Museum of China (**NZMC**), Institute of Zoology, Chinese Academy of Sciences, Beijing, China. Additionally, two slides of types were loaned from Taiwan Agricultural Research Institute (**TARI**), Taiwan, China.

## ﻿Taxonomy

### ﻿ Azaleothrips

Taxon classificationAnimaliaThysanopteraPhlaeothripidae

Ananthakrishnan, 1964

76DCDD83-D711-5294-9B99-3FD9ABE7A506

Azaleothrips Ananthakrishnan, 1964: 220. Type species AzaleothripsamabilisAnanthakrishnan 1964, by monotypy.

#### Note.

The number of sense cones on antennal segments III–IV is a very diagnostic character in many thysanopteran taxa. Especially in *Azaleothrips*, two species groups were proposed because of two sense cones on III and two or three cones on IV in the *moundi* species group, and three and four on antennal segments III–IV, respectively, in the *amabilis* species group. These numbers vary between species, but are stable within each species. Furthermore, the macropterous forms in this genus are very common, but three species also have micropterae which usually bear three well-developed sub-basal setae, *A.moundi*, *A.simulans* and *A.sphaericus* sp. nov., that all belong to *moundi* species group. The complex sculptures on the body surface are also various ranged from weakly reticulate to strongly reticulate with wrinkles or tubercles inside or along lines. Therefore, these variations are not helpful to give a clear generic diagnosis, but fortunately an excellent one is available in a recent paper (Okajima and Masumoto 2014).

### ﻿Key to species of *Azaleothrips* from China

*A.taiwanus* and *A.atayal* are included in the key based on the excellent original descriptions.

**Table:** 

1	Antennal segment III with two sense cones	**2**
–	Antennal segment III with three sense cones	**3**
2	Antennal segment IV with two sense cones (Fig. 11)	***A.moundi***
–	Antennal segment IV with three sense cones (Fig. 13)	***A.sphaericus* sp. nov.**
3	Fore-femora yellow to pale brown, much paler than head	**4**
–	Fore-femora brown to dark brown, as dark as head	**6**
4	Metanotum longitudinally reticulate on anterior half, and with polygonal reticulations on posterior half (Fig. 23)	***A.templeri***
–	Metanotum longitudinally reticulate or striate, and without polygonal reticulations	**5**
5	S1 setae on abdominal tergite IX slightly shorter than half the length of tube in both sexes	***A.lepidus***
–	S1 setae on abdominal tergite IX longer than half the length of tube in both sexes	***A.formosae***
6	Pronotum yellowish brown, at least paler than head	***A.siamensis***
–	Pronotum uniformly brown, as dark as head	**7**
7	Head sculptured with very weak lines of reticulation (Fig. 2); mouth-cone long and sharply pointed that reaches to mesopresternum (Fig. 3); S2 on tergite IX sharply pointed at apex in both sexes (Fig. 29)	***A.laevigatus***
–	Head strongly sculptured with reticulation; mouth-cone moderately long that reaches to posterior margin of ferna at most; S2 on tergite IX expanded at apex in female	**8**
8	Pronotum sculptured with lines of reticulation as well as many small tubercles along lines	***A.taiwanus***
–	Pronotum finely reticulated, but almost smooth along reticles, without tubercles	**9**
9	Tergite IX setae S1 much shorter than half of tube length; antennal segment III clearly yellow (Fig. 9)	***A.laocai***
–	Tergite IX setae S1 almost as long as half of tube length; antennal segment III somewhat shaded at apical half	***A.atayal***

### ﻿ Azaleothrips atayal

Taxon classificationAnimaliaThysanopteraPhlaeothripidae

Okajima & Masumoto, 2014

A3A1ADB0-774C-52F4-8E10-47031242CF69

Azaleothrips atayal Okajima & Masumoto, 2014: 309.

#### Comments.

Described from Taiwan on dead leaves and branches, this species is a member of the *amabilis* species group, as indicated by Okajima and Masumoto (2014). *Azaleothripsatayal* is closely related to another Taiwan species, *A.formosae*, which can be distinguished from *A.formosae* by its darker body; it has also been collected at lower altitudes, as mentioned by Okajima and Masumoto (2014). Unfortunately, no specimen was studied here, but the species is readily placed in the above key using the excellent illustrated description.

### ﻿ Azaleothrips formosae

Taxon classificationAnimaliaThysanopteraPhlaeothripidae

Okajima & Masumoto, 2014

98DFE64D-D750-5BA9-BE79-ECC8382DE699

Figs 1 , 8 , 15 , 27

Azaleothrips formosae Okajima & Masumoto, 2014: 320.

#### Specimens studied.

China – **Yunnan** • 1♀ (SNUT); Puer, Zhenyuan; on dead leaves; 8.vii.2022; Yanqiao Li leg. • 1♂ (SNUT); Lincang, Cangyuan; on dead leaves; 8.vi.2021; Xia Wang & Chengwen Li leg. • 1♂ (ANIC); Kunming; on bamboo grass; 24.ix.2019; Laurence Mound leg.

#### Comments.

This species was described on many specimens, including types and non-types, from Taiwan that were collected on dead branches. A female and two males from Yunnan are identified as *A.formosae* because they show no difference in their morphology. However, they differ in their coloration; the female has the prothorax largely yellow, like non-paratypic specimens from Kenting National Park (Taiwan), and one male has a brownish prothorax (Fig. 15), slightly paler than the head, and all femora yellowish brown with the apical quarter pale; the other male has a pale prothorax, like the paratypes. Moreover, the pore plate on abdominal sternite VIII is a little broader in the Yunnan specimens (Fig. 27), but S2 setae on tergite IX are pointed at the apex, as in the types.

**Figures 24–30.**
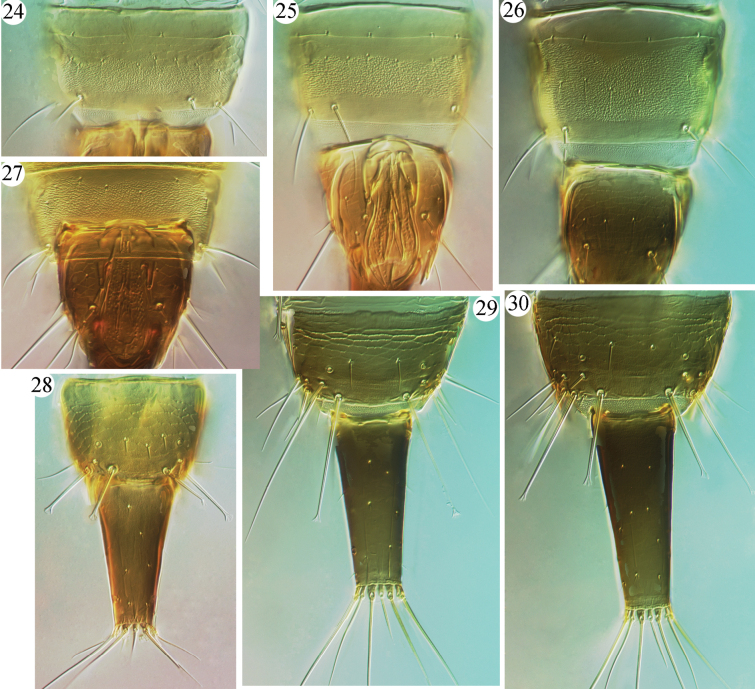
Abdomen of *Azaleothrips* species **24–27** pore plate on sternite VIII **24***A.sphaericus* sp. nov. **25***A.laevigatus***26***A.lepidus***27***A.formosae***28–30** tergites IX–X **28***A.sphaericus* sp. nov. **29***A.laevigatus***30***A.siamensis*.

### ﻿ Azaleothrips laevigatus

Taxon classificationAnimaliaThysanopteraPhlaeothripidae

Okajima, 2006

31990243-74D2-5AFA-BA85-2E953F14DD45

Figs 2 , 3 , 10 , 16 , 25 , 29

Azaleothrips laevigatus Okajima, 2006: 192.

#### Specimen studied.

China – **Guangxi** • 1♀1♂ (SNUT); Chongzuo; on dead wood; 9 & 25.vii.2021; Xia Wang leg.

#### Comments.

Described from Japan on dead *Casuarina* branches, this species is distinguished easily from other *Azaleothrips*, except for two Philippine species, *A.philippinensis* and *A.bifidius*, in having S2 on abdominal tergite IX of males pointed at its apex (Fig. 29). *Azaleothripslaevigatus* can be distinguished from these Philippine species by the weaker sculpture on the head and pronotum and transverse pore plate on male sternite VIII (Figs 2, 16, 29). This species is closely related to an Indonesian species, *A.dentatus*, in having weak sculpture on the body surface and shorter major setae, and in the head shape, but in *A.dentatus* the fore-tibia has an apical inner tubercle. Additionally, the head of *A.laevigatus* has weak sculpture, almost straight cheeks, postocular setae close together and slender, and long stylets that reach the eyes (Fig. 2), similar to species of *Ablemothrips*. However, *A.laevigatus* has a long mouth-cone which is sharply pointed and reaching the mesopresternum (Fig. 3), and the postocular setae are also close together in male, while *Ablemothrips* species have the mouth cone-short and rounded, and the postocular setae of males are sexually dimorphic and widely separated (Okajima 1999). A female and male from Guangxi, China are recognized as *A.laevigatus* because there is no differences in morphology and coloration, as compared to the original description (Okajima 2006).

### ﻿ Azaleothrips laocai

Taxon classificationAnimaliaThysanopteraPhlaeothripidae

Okajima & Masumoto, 2014

59C41362-B848-552C-8264-BBF2D9D066DC

Fig. 9

Azaleothrips laocai Okajima & Masumoto, 2014: 325.

#### Specimens studied.

China – **Shaanxi** •1♀ (SNUT); Hanzhong; on dead leaves; 20.vii.2017; Lihong Dang leg. • 1♀ (SNUT); Yanan; 25.vii.2019; Weiyan Liu leg.

#### Comments.

*Azaleothripslaocai* was described from Vietnam on dead branches. It belongs to the *amabilis* species group, which bears three and four sense cones on antennal segments III and IV, respectively. Currently, this species has the largest body size of any known *Azaleothrips* species, with two females from Shaanxi, China about 2270–2350 μm in body length, whereas the body lengths of other *Azaleothrips* spcies are usually no more than 2000 μm. These two females from Shaanxi show a little difference in antennae coloration; segment IV is brown, with the apex and base pale, as well as the base of V pale (Fig. 9), but in the original description of *A.laocai* the basal neck of IV is yellowish and V is uniformly brown.

### ﻿ Azaleothrips lepidus

Taxon classificationAnimaliaThysanopteraPhlaeothripidae

Okajima, 1978

3291D832-2508-5469-B49D-C5E0501B0274

Figs 5 , 17 , 26

Azaleothrips lepidus Okajima, 1978: 386.

#### Specimens studied.

China – **Yunnan** • 1♀1♂ (SNUT); Lincang; on dead branches; 4 & 7.vi.2021; Xia Wang & Chengwen Li leg. LAOS – **Champasak** • 4♀1♂ (ANIC); on dead wood; 12.vi.2018; Alice Wells leg.

#### Comments.

Described from Thailand on dead leaves, this species is here newly recorded from China. *Azaleothripslepidus* is very similar to *A.toshifumii*, with which it is sympatric, but they can be distinguished by the coloration of antennae and legs (Okajima and Masumoto 2014). Two specimens from China have antennal segment IV uniformly brown, fore-coxae and femora largely yellow, with outer side shaded, and the mid- and hind-tibiae slightly medially shaded. Especially, their antennal segment III is clear yellow, as are the specimens from Laos, which were originally described as yellow to yellowish brown (Okajima 1978).

### ﻿ Azaleothrips moundi

Taxon classificationAnimaliaThysanopteraPhlaeothripidae

Okajima, 1976

0BD2FD07-253B-5895-84D2-C0562399D82A

Fig. 11

Azaleothrips moundi Okajima, 1976: 19.

#### Specimens studied.

China – **Taiwan** • 2♀ (TARI, female holotype and paratype of ‘*magnus*’); Taipei, Hsien; on dead twigs of *Morusaustralis*; 12.viii.1978; Liansheng Chen leg. **Sichuan** • 1♀ (SNUT); Chengdu; on dead leaves; 14.viii.2021; Xin Li leg. **Shanxi** • 1♂ (SNUT); Jincheng, Mishui; 16.vi.2022; Yuxin Gao leg.

#### Comments.

This species, one of the two species from China that belong to the *moundi* species group, was described from Japan and Taiwan on dead twigs. It is unique in *Azaleothrips* because antennal segments III–IV both have two major sense cones (Fig. 11). The holotype female and paratype male of *A.magnus* Chen, 1980, which was synonymized with *A.moundi* by Okajima and Masumoto (2014), were here studied, but both specimens had crack inside the balsam that seems to have allowed entry of air under the cover slip. These specimens have the third antennal segment III clear yellow, as in the original description of *A.moundi*. Two micropterous female and male from Sichuan and Shanxi recognized here as *A.moundi* have antennal segment III shaded on apical half. This was also noted for *A.Lepidus*, as mentioned above.

### ﻿ Azaleothrips siamensis

Taxon classificationAnimaliaThysanopteraPhlaeothripidae

Okajima, 1978

494CFE5D-314F-5150-B446-9B9F0567F672

Figs 6 , 12 , 18 , 30

Azaleothrips siamensis Okajima, 1978: 389.

#### Specimens studied.

China – **Guangxi** • 1♀ (SNUT); Chongzuo, Daxin; on dead wood; 25.vii.2021; Xia Wang leg. **Yunnan** • 4♀1♂ (NZMC); Mengla; one dead branch; 10 & 17.iv.1997; Yunfa Han leg. **Chongqing** • 1♀ (NZMC); one dead branch; 1.viii.1999; Yunfa Han leg.

#### Comments.

Described from northern Thailand on dead leaves, this species was first recorded from China, Guizhou, by Han (1992). Here, six females and one male each from Guangxi, Chongqing, and Yunnan suggest that this species may be common in southern China. These specimens have much paler pronotum (Fig. 18) and shorter anal setae than the specimens described from Thailand in the original description (Okajima 1978), but no other differences have been observed.

### ﻿ Azaleothrips sphaericus sp. nov.

Taxon classificationAnimaliaThysanopteraPhlaeothripidae

5FE6DDB5-03F7-5842-AAC0-F21C213B95E3

https://zoobank.org/43025455-5059-4D41-82A1-7E538200640D

Figs 4 , 13 , 19–21 , 24 , 28

#### Specimens studied.

***Holotype***, China – **Guangxi** • ♀ macroptera (SNUT); Chongzuo, Fusui; on dead branch; 8.viii.2021; Xia Wang leg. ***Paratypes***, China – **Guangxi** • 1♀ macroptera (SNUT); Nanning, Wuming; 18.vii.2021; Xia Wang leg. • 3♀ microptera (SNUT, ANIC); Chongzuo, Daxin; 25.vii.2021; Xia Wang leg. • 1♂ microptera (SNUT); Nanning, Shanglin; 18.vii.2021; Xia Wang leg.

#### Description.

***Holotype*. Female macroptera.** Body uniform brown. Antennal segment III clear yellow (Fig. 13); other segments brown, but I–II slightly lighter than head. All femora brown, with apices and extreme bases yellowish; all tibiae brown at middle, with apices and bases yellowish; mid- and hind-tibiae sightly paler. Fore-wing shaded with brown, paler in basal quarter.

***Head*** (Fig. 4). Head distinctly longer than wide; dorsal surface strongly sculptured with reticles, with scattered small wrinkles among reticles, especially between postocular setae. Compound eyes comparatively small, about 0.3 times as long as head. Postocular setae shorter than half length of eyes. Antennal segments VII and VIII tightly fused (Fig. 13); median segments spherical, III smaller than IV; segment III with two (1 + 1), segment IV with three (1 + 2) sense cones.

***Thorax*** (Figs 19, 20). Pronotum distinctly sculptured with rows of small tubercles, but median portion with distinctive circular sculpture; pronotum with 26 short setae (Fig. 19). Basantra present, but weak. Mesonotum with small, dentate microtrichia or tubercles along transverse lines of sculpture, almost smooth between lines (Fig. 20). Metanotum with polygonal reticulations, with delicate wrinkles within reticles, and with small tubercles along lines of reticles on posterior quarter (Fig. 20); anterior half with 15 short setae, including three pairs in anterior angles. Mesopresternum boat-shaped but narrowed laterally. Metathoracic sternopleural suture present. Fore-tarsus unarmed. Fore-wing contracted medially with 4/7 duplicated cilia; subbasal setae S3 longer than S1 and S2, but much shorter than the contracted portion of fore-wing.

***Abdomen*** (Figs 24, 28). Pelta distinctly medially reticulate, with delicate wrinkles within reticles (Fig. 20). Abdominal tergites II–VII weakly sculptured with transverse reticles or lines, dentate microtrichia in lateral third, with two pairs of wing-retaining setae; posterior pair much larger than anterior pair. Tergite VIII with a pair of small setae medially; tergite IX with four short setae at middle (Fig. 28); S1 setae on tergite IX a little shorter than half length of tube, expanded at apex (Fig. 28); S2 slightly longer than S1, expanded at apex (Fig. 28); S3 as long as tube, pointed at apex. Tube short, about 0.6 times as long as head. Anal setae longer than tube.

Measurements (holotype female in μm). Body length 1580. Head length 170, width across cheeks 165. Compound eye dorsal length 55. Pronotum length 110, width 200. Fore wing length 550. Tube length 105, width across base 55, apical width 30. Antenna length 270, segments I–VIII length (width) as follows: 30 (30), 40 (30), 35 (28), 40 (28), 37 (25), 37 (25), 30 (20), 20 (15). Postocular setae about 23. S1–S3 on tergite IX 43, 53, 115. Anal setae 115.

Female microptera. Color and structure similar to macropterous female, but tubercles on lines of reticles in most part of head, metanotum, pelta, and posterior median portion of tergites II–VII; metanotum and pelta broad (Fig. 21); eyes and two pairs of wing-retaining setae relatively small.

Measurements (paratype micropterous female in μm). Body length 1470. Head length 180, width across cheeks 170. Compound eye dorsal length 45. Pronotum length 125, width 210. Fore wing length 125. Tube length 100, width across base 55, apical width 30. Antenna length 305, segments I–VIII length (width) as follows: 30 (30), 42 (30), 35 (27), 40 (27), 40 (27), 40 (27), 27 (20), 25 (17). Postocular setae about 25. S1–S3 on tergite IX 37, 67, 110. Anal setae 125.

Male microptera. Color and structure very similar to micropterous female, but mid- and hind-tibiae yellow with slightly shaded medially. Pore plate on abdominal sternite VIII distinct, narrow (Fig. 24). S2 on abdominal tergite IX expanded at apex, slightly longer than S1.

Measurements (paratype micropterous male in μm). Body length 1350. Head length 170, width across cheeks 155. Compound eye dorsal length 45. Pronotum length 120, width 185. Fore wing length 100. Tube length 95, width across base 50, apical width 25. Antenna length 260, segments I–VIII length (width) as follows: 27 (27), 35 (27), 30 (27), 35 (25), 35 (25), 35 (25), 27 (20), 22 (15). Postocular setae 20. S1–S3 on tergite IX 35, 40, 95. Anal setae 105.

#### Etymology.

Latin, *sphaericus*, referring to the spherical antennal segment III.

#### Comments.

This new species is unusual among *Azaleothrips* species in having antennal segment III almost spherical and smaller than IV, a condition somewhat similar with some *Strepterothrips* species. The new species is similar to *A.phuketanus* and *A.simulans* in having two and three sense cones on antennal segments III and IV, respectively, but it differs from *A.phuketanus* in having antennal segment III shorter than IV (Fig. 13), the anterior part of metanotum with 15 small setae before the major pair of setae, whole of pronotum covered with tubercles (Figs 19, 20), and S1 setae of tergite IX shorter than one-half the length of the tube (Fig. 28); in *A.phuketanus* antennal segment III is much longer than IV, the anterior part of metanotum bears seven small setae before the major pair of setae, the pronotum only has tubercles posterior to median, and S1 setae of tergite IX is longer than half the length of tube. It is distinguished from *A.simulans* in having antennal segments I–II concolorous with the head, VI uniformly brown, antennal segment III shorter than IV (Fig. 13), the dorsal surface of head reticulate and without an asperate area (Fig. 4), the metanotum with wrinkles among reticles and the anterior part of metanotum with 15 small setae before the major pair of setae (Fig. 20), and a hat-shaped pelta; in *A.simulans* antennal segments I and II are paler than the brown head, the basal neck of VI is yellow, antennal segment III much longer than IV, the dorsal surface of the head is reticulate but medially is aspirate and not reticulate, the metanotum is strongly asperate and without lines of reticulation at centrally, the anterior part of metanotum has at most 10 small setae before the major pair of setae, and the pelta is trapezoid. It is also similar to *A.moundi* in having antennae brown and only with III yellow and with two sense cones, in sculpture of the head and pronotum, and setae on tergite IX, but these species can be distinguished as follows. *Azaleothripssphaericus* has antennal segment IV with three major sense cones, antennal segment III spherical, with a short stem (Fig. 13) which is almost as long as wide, antennal segment IV uniformly brown, postocular setae about half length of the eyes (Fig. 4), apices and bases of all tibiae pale, light brown, and pelta with tubercles on lines of transverse reticulation in microptera (Fig. 21); in *A.moundi* antennal segment IV has two major sense cones, antennal segment III is not spherical and longer than wide, basal neck of IV is paler, postocular setae shorter than one-half length of the eyes, all tibiae brown to dark brown, apices and bases of mid- and hind-tibia pale, and pelta polygonally reticulate in microptera.

### ﻿ Azaleothrips taiwanus

Taxon classificationAnimaliaThysanopteraPhlaeothripidae

Okajima & Masumoto, 2014

41D9ADD4-9BAA-5DF6-8BDD-2E1DA30C19B8

Azaleothrips taiwanus Okajima & Masumoto, 2014: 342.

#### Comments.

Described from Taiwan on dead branches, this species was not satisfactorily distinguished from another Taiwan species, *A.atayal*, in the original description (Okajima and Masumoto 2014). The only difference between these two species is the presence or absence of many small tubercles in the reticulation of head and pronotum, as indicated in the above key.

### ﻿ Azaleothrips templeri

Taxon classificationAnimaliaThysanopteraPhlaeothripidae

Okajima & Masumoto, 2014

C49F4FDC-E734-5183-8FC6-F3F4A2CC6998

Figs 7 , 14 , 22 , 23

Azaleothrips templeri Okajima & Masumoto, 2014: 342.

#### Specimens studied.

China – **Guangxi** •1♀ (SNUT); Qianzhou, Shiwandashan; 5.viii.2015; Chunfeng Li leg. LAOS – **Champasak** • 1♂ (ANIC); on dead leaves of Cordyline; 12.vi.2018; Alice Wells leg.

#### Comments.

Described from West Malaysia on dead leaves, this species is similar to *A.lepidus* in having pale pronotum (Fig. 22) and fore-femora, but *A.templeri* can be easily distinguished from other Chinese species of *Azaleothrips*. It is distinguished by the presence of polygonal reticulation on the posterior half of the dorsal surface of metathorax (Fig. 23). A female from Guangxi is identified as *A.templeri*, and this is the first record of the species from China. A male from Laos is also recognized here as this species.

## Supplementary Material

XML Treatment for Azaleothrips

XML Treatment for Azaleothrips atayal

XML Treatment for Azaleothrips formosae

XML Treatment for Azaleothrips laevigatus

XML Treatment for Azaleothrips laocai

XML Treatment for Azaleothrips lepidus

XML Treatment for Azaleothrips moundi

XML Treatment for Azaleothrips siamensis

XML Treatment for Azaleothrips sphaericus

XML Treatment for Azaleothrips taiwanus

XML Treatment for Azaleothrips templeri

## ﻿Additional information

### Author ORCIDs

Lihong Dang https://orcid.org/0000-0002-7571-8426

Yaya Li https://orcid.org/0009-0009-3227-5969

Laurence A. Mound https://orcid.org/0000-0002-6019-4762

Gexia Qiao https://orcid.org/0000-0002-7300-6812

### Data availability

All of the data that support the findings of this study are available in the main text.
